# The distribution of *jhp0940*, *jhp0945*, *jhp0947*, *jhp0949* and *jhp0951* genes of *Helicobacter pylori* in China

**DOI:** 10.1186/s12876-015-0341-z

**Published:** 2015-09-10

**Authors:** Yanan Gong, Xianhui Peng, Lihua He, Hao Liang, Yuanhai You, Jianzhong Zhang

**Affiliations:** 1 State Key Laboratory of Infectious Disease Prevention and Control, National Institute for Communicable Disease Control and Prevention, Chinese Center for Disease Control and Prevention, 155 Changbai Road, Changping District, Beijing, 102206 China; 2 Collaborative Innovation Center for Diagnosis and Treatment of Infectious Diseases, Hangzhou, China

## Abstract

**Background:**

The plasticity region of *Helicobacter pylori* (*H. pylori*) is a large chromosomal segment containing strain-specific genes. The prevalence of the plasticity region genes of the *H. pylori* strains in China remains unknown. The aim of this study was to examine the status of these genes and to assess the relationship between the genes and the diseases caused by *H. pylori* infection.

**Methods:**

A total of 141 strains were isolated from patients with chronic active gastritis (CAG), peptic ulcer disease (PUD) and gastric carcinoma (GC). The prevalence of *jhp0940*, *jhp0945*, *jhp0947*, *jhp0949* and *jhp0951* was determined using PCR, and the results were analyzed using the chi-squared test.

**Results:**

The prevalence rates of *jhp0940, jhp0945, jhp0947, jhp0949* and *jhp0951* in the *H. pylori* strains were 42.55, 51.06, 20.57, 56.03 and 63.12 %, respectively. The prevalence rates of *jhp0940* were similar in the isolates from the CAG, PUD and GC patients, and there was no association between the *jhp0940* status and any of the diseases. In contrast, the prevalence rates of *jhp0945*, *jhp0947*, *jhp0949* and *jhp0951* were significantly higher in the PUD and GC isolates than in the CAG isolates (*p* < 0.01). A univariate analysis showed that *jhp0945*, *jhp0947*, *jhp0949* and *jhp0951* increased the risk of PUD, while only *jhp0951* was significantly associated with PUD in the multivariate analysis (*p* = 0.0149). The *jhp0945*-positive isolates were significantly associated with an increased risk for GC (*p* = 0.0097).

**Conclusion:**

The plasticity region genes are widely distributed in Chinese patients, and a high prevalence of these genes occurs in more serious diseases. Therefore, *jhp0951* status is an independent factor associated with the development of PUD, and *jhp0945* may predict the future development of GC in patients with CAG and is considered to be the best candidate disease marker for *H. pylori*-related diseases.

## Background

*H. pylori* is the major cause of chronic gastritis, peptic ulcer disease, gastric carcinoma and mucosa-associated lymphoid tissue lymphoma [1, 2]. The majority of infected individuals remain asymptomatic throughout their lifetime, and only approximately 15 % develop gastroduodenal diseases. Variations in the clinical outcomes of these diseases have been attributed to differences in environmental factors, bacterial strains and host genetics [3, 4]. A number of bacterial virulence factors that are associated with these diseases have been described for *H. pylori*, such as the *cag* pathogenicity island (*cag* PAI) [5, 6].

A comparison of the genomes of two *H. pylori* strains revealed that in addition to the *cag* PAI, a large region of approximately 45 kb in strain J99 and 68 kb in strain 26695 is present in both strains and has been termed the “plasticity region” [7, 8] . Up to 50 % of the strain-specific genes transferred from other species are located in the plasticity region [9]. Whether these strain-specific genes influence the severity of different diseases or the biological functions of the ORFs in the plasticity region still remains unknown. Recent studies have revealed that the *jhp0940*, *jhp0945*, *jhp0947*, *jhp0949* and *jhp0951* genes from *H. pylori* are associated with an increased risk for gastroduodenal diseases [3, 9–11]. In a Brazilian study, the *jhp0947* gene was found to be involved in the development of duodenal ulcers (DUs) and GC [11]. In addition, Romo-González et al. found that *jhp0951* is also associated with DUs [9]. In China, the number of *H. pylori* infections has exceeded 60 %, and the high-risk incidence of GC poses a serious health and economic burden. Moreover, there are no reports discussing the prevalence of these genes or the relationship between the genes and the severity of the clinical outcomes of the abovementioned diseases in China. The aim of this study was to assess the prevalence of *jhp0940*, *jhp0945*, *jhp0947*, *jhp0949* and *jhp0951* and to determine their association with *H. pylori*-related diseases.

## Methods

### Strains

A total of 141 *H. pylori* strains were selected from the *H. pylori* strain bank of China at the National Institute for Communicable Disease Control and Prevention, Chinese Center for Disease Control and Prevention. A total of 40 strains were isolated from patients in Heilongjiang (HLJ) Province (located in northern China), and another 101 strains were isolated from patients from Jiangxi (JX) Province (located in southeast China). These strains were related with CAG only (*n* = 58), PUD (*n* = 45) or GC (*n* = 38). Two strains with fully sequenced genomes, including strain 26695, which was isolated from a gastritis patient, and strain J99, which was isolated from a patient with a duodenal ulcer, were used as controls. This study was approved by the Ethics Committee of National Institute for Communicable Disease Control and Prevention, Chinese Center for Disease Control and Prevention. The written informed consent was obtained from all patients.

### Culture and extraction of genomic DNA

The strains stored in brain heart infusion in −80 °C were recovered on Columbia agar plates (Oxoid) supplemented with 5 % fresh defibrinated sheep blood and kept under microaerophilic conditions (5 % O2, 10 % CO2 and 85 % N2) at 37 °C for 3 days. Colonies displaying typical *H. pylori* morphology were selected and identified by Gram staining and urease, oxidase, and catalase activity testing. The bacterial cells on chocolate agar plate were washed twice with phosphate buffer saline (PBS, pH7.5) and centrifuged at 5000 rpm for 10 min. The chromosomal DNA was extracted using the QIAamp DNA Mini Kit (Qiagen, Germany) according to the manufacturer’s instructions.

### Determination of the *jhp0940, jhp0945, jhp0947, jhp0949* and *jhp0951* status via PCR

The status of the genes was determined via PCR using the primer pairs shown in Table 1. The amplification of the genes was performed in a volume of 25 μl containing 25 pmol of both forward and reverse primers. The PCR conditions were 95 °C for 5 min, followed by 35 cycles of 95 °C for 45 s, 52 °C for 45 s, and 72 °C for 45 s, and finally 72 °C for 5 min. Two primer sets were used for the *jhp0940* gene, and when the PCR using one pair was positive, the *jhp0940* status was determined to be positive. The status of the genes was positive in strain J99 and negative in strain 26695.

**Table 1:** The primer sequences for the five genes

Primer name	Primer sequence (5′-3′)	Product size	Reference
*jhp0940-1*	F5′-GAAATGTCCTATACCAATGG	591 bp	[3]
	R5′-CCTAAGTAGTGCATCAAGG		
*jhp0940-2*	F5′-ATGCCAACCATTGATTTTACTTTT	978 bp	[12]
	R5′-TTATCGTCTACGCTTAGGTGTG		
*jhp0945*	F5′-CAATGCGACTAACAGCATAG	1028 bp	[3]
	R5′-CGCATTTGCTGTCATCTTTG		
*jhp0947*	F5′-GATAATCCTACGCAGAACG	611 bp	[3]
	R5′-GCTAAAGTCATTTGGCTGTC		
*jhp0949*	F5′-TTCAAAAAGTCCCCGAAATG	235 bp	[10]
	R5′-GGATGTCCTGGCATGTCTCT		
*jhp0951*	F5′-ATGCGTGGCTAAGCGATACT	243 bp	[19]
	R5′-GACCCAACGCTCTTGAAGTT		

The primer sequences used for the five genes were the same as the references. There were two pairs of primers used for *jhp0940*

F, forward primer sequence; R, reverse primer sequence

### Statistical analysis

The prevalence rates of all five genes were evaluated. The association between each genotype and the clinical outcomes were quantified using the chi-squared test. A probability (*p*) value equal to or less than 0.05 was considered to be statistically significant, and a value of 0.001 or less was considered to be highly significant. Univariate analysis and a multivariate logistic regression model were used to calculate the odds ratios (ORs) of the clinical outcomes using SAS 9.3.

## Results

### Distribution of the *jhp0940, jhp0945, jhp0947, jhp0949* and *jhp0951* genes

The prevalence rates of *jhp0940, jhp0945, jhp0947, jhp0949* and *jhp0951* in the tested *H. pylori* were 42.55 % (60/141), 51.06 % (72/141), 20.57 % (29/141), 56.03 % (79/141) and 63.12 % (89/141), respectively (Table 2).

**Table 2:** The prevalence of the five genes in the CAG, PUD and GC isolates

		No^a^ (%) found			
	Gene	CAG^b^	PUD^c^	GC^d^	Total^e^
		HLJ (*n* = 20)	HLJ (*n* = 9)	HLJ (*n* = 11)	
		JX (*n* = 38)	JX (*n* = 36)	JX (*n* = 27)	
HLJ^f^	*jhp0940*	6 (30 %)	3 (33.33 %)	7 (63.64 %)	16 (40 %)
*n* = 40					
	*jhp0945*	5 (25 %)	4 (44.44 %)	8 (72.73 %)	17 (42.5 %)
	*jhp0947*	2 (10 %)	0	2 (18.18 %)	4 (10 %)
	*jhp0949*	8 (40 %)	7 (77.78 %)	6 (54.55 %)	21 (52.5 %)
	*jhp0951*	11 (55 %)	6 (66.67 %)	6 (54.55 %)	23 (57.5 %)
JX^g^	*jhp0940*	14 (36.84 %)	19 (52.78 %)	11 (40.74 %)	44 (43.56 %)
*n* = 101					
	*jhp0945*	11 (28.95 %)	24 (66.67 %)	20 (74.07 %)	55 (54.46 %)
	*jhp0947*	2 (5.26 %)	15 (41.67 %)	8 (29.63 %)	25 (24.75 %)
	*jhp0949*	15 (39.47 %)	24 (66.67 %)	19 (70.37 %)	58 (57.43 %)
	*jhp0951*	20 (52.63 %)	29 (80.56 %)	17 (62.96 %)	66 (65.35 %)

The positive rates of each gene in the CAG, PUD and GC patients from HLJ and JX Provinces

^a^*No* the number of positive isolates

^b^*CAG* chronic active gastritis

^c^*PUD* peptic ulcer disease

^d^*GC* gastric carcinoma

^e^*Total* the total positive rate of each gene

^f^*HL* Heilongjiang Province, China

^g^*JX* Jiangxi Province, China

### Geographic variation of plasticity region genes

The results were divided into two groups based on their geographic variation. By comparing the results of the strains from HLJ Province and JX Province, we found no significant differences in the prevalence of *jhp0940* (40 %, 16/40 and 43.56 %, 44/101, respectively, *p* = 0.699), *jhp0945* (42.5 %, 17/40 and 54.46 %, 55/101, *p* = 0.201), *jhp0947* (10 %, 4/40 and 24.75 %, 25/101, *p* = 0.051), *jhp0949* (52.5 %, 21/40 and 57.43 %, 58/101, *p* = 0.595), or *jhp*0951 (57.5 %, 23/40 and 65.35 %, 66/101, *p* = 0.384).

### Plasticity region genes and diseases

The prevalence rates of *jhp0940* in the strains from the CAG, PUD and GC patients were 34.48 % (20/58), 48.89 % (22/45) and 47.37 % (18/38), respectively, and there were no differences between the three diseases. The prevalence rates of *jhp0945* in the isolates from the patients with PUD (62.22 %, 28/45) and GC (73.68 %, 28/38) were significantly higher than from the CAG patients (27.59 %, 16/58; *p* = 0.0004 and *p* < 0.0001, respectively). The *jhp0947* gene occurred in only 4 (6.89 %) of the strains isolated from the CAG patients, and the occurrence was much lower than in the PUD and GC patients (33.33 %, 15/45 and 26.32 %, 10/38; *p* = 0.0006 and *p* = 0.0084, respectively). The prevalence of *jhp0949* was similar (68.89 %, 31/45 and 65.79 %, 25/38) in the PUD and GC isolates and was much higher than that in the CAG patients (39.66 %, 23/58; *p* = 0.0032 and *p* = 0.0123, respectively). For *jhp0951*, the prevalence rate in the PUD patients was higher than that in the CAG patients (77.78 %, 35/45 and 53.45 %, 31/58, respectively; *p* = 0.0107); however, there was no difference between GC and CAG.

### The relationship between *jhp0940, jhp0945, jhp0947, jhp0949* and *jhp0951*

The status of all of the genes showed that they are significantly associated with each other (Table 3). The status of *jhp0945* was associated with *jhp0949* (*p* = 0.009) and *jhp0951* (*p* = 0.022). All of the *jhp0947*-positive isolates possessed *jhp0949*, and both were significantly associated with *jhp0951* (*p* = 0.0008 and *p* < 0.0001, respectively).

**Table 3:** The relationship between the different plasticity region genes

	Relationship (coefficient value^a^/*p* value)			
gene	*jhp0945*	*jhp0947*	*jhp0949*	*jhp0951*
*jhp0940*	0.183/0.03	0.236/0.005	0.184/0.029	0.182/0.031
*jhp0945*	—	0.358/<0.0001	0.219/0.009	0.193/0.022
*jhp0947*	—	—	0.415/<0.0001	0.280/0.0008
*jhp0949*	—	—	—	0.478/<0.0001

The coefficient value for assessing the association between each pair of genes. A *p* value of 0.001 or less was considered to be highly significant

The coefficient value^a^ was analyzed using Fisher’s test

When combining the five genes together, the majority of the genotypes were all negative or all positive (the −/−/−/−/- genotype or the +/+/+/+/+ genotype, 12.06 %, 17/141). The rates of the all-positive genotypes were 15.79 % (6/38), 20 % (9/45) and 3.45 % (2/58) in the isolates from the patients with GC, PUD and CAG only, respectively. In contrast, the rates of the all-negative genotypes were 5.26 % (2/38), 2.22 % (1/45) and 24.14 % (14/58) for GC, PUD, and CAG, respectively.

### The relationship between the gene status and the clinical outcomes

A univariate analysis showed that there was no significant association between the *jhp0940* status and the selected diseases (Table 4), but the status of *jhp0945*, *jhp0947*, *jhp0949* and *jhp0951* was significantly associated with a lower risk for CAG (odds ratio (OR), [95 % confidence interval (CI)], 0.176 [0.081 to 0.381]; 0.116 [0.035 to 0.389]; 0.300 [0.145 to 0.623] and 0.445 [0.214 to 0.928], respectively). On the converse, they were also related to an increased risk for PUD (OR [95 % CI], 2.306 [1.08 to 4.927]; 2.835 [1.191 to 6.751], 2.452 [1.126 to 5.336] and 2.825 [1.226 to 6.51], respectively). Moreover, only the *jhp0945*-positive isolates were associated with an increased risk for GC (OR, 5.9259; 95 % CI, 1.267 to 27.714).

**Table 4:** The relationship between each gene and the clinical outcomes

		CAG^a^			PUD^b^		GC^c^		
gene	OR^d^	95 % CI^e^	*p*	OR	95 % CI	*p*	OR	95 % CI	*p*
*jhp0940*	0.488	0.235–1.013	0.054	1.449	0.694–3.026	0.323	1.822	0.734–4.523	0.196
*jhp0945*	0.176	0.081–0.381	<0.0001	2.306	1.08–4.927	0.031	3.542	1.362–9.209	0.009
*jhp0947*	0.116	0.035–0.389	0.0005	2.835	1.191–6.751	0.019	2.307	0.765–6.952	0.134
*jhp0949*	0.300	0.145–0.623	0.0012	2.452	1.126–5.336	0.024	2.067	0.812–5.26	0.128
*jhp0951*	0.445	0.214–0.928	0.031	2.825	1.226–6.51	0.015	1.008	0.403–2.517	0.987

The univariate analysis showing the association between each gene status and the indicated disease

^a^*CAG* chronic active gastritis

^b^*PUD* peptic ulcer disease

^c^*GC* gastric carcinoma

^d^*OR* odds ratio

^e^*CI* confidence interval

A multivariate analysis, including age and *jhp0940, jhp0945, jhp0947, jhp0949* and *jhp0951* status, was performed to determine the factors that were related to the clinical outcomes of the selected diseases (Table 5). The *jhp0945* status for GC and the *jhp0951* status for PUD significantly increased the risk of a clinical outcome. In contrast, the *jhp0945* or *jhp0949* status significantly decreased the risk for CAG (OR [95 % CI]: 0.214 [0.099 to 0.466]; 0.373 [0.172 to 0.807], respectively). Age significantly decreased the risk of CAG and PUD (OR [95 % CI]: 0.964 [0.937 to 0.992]; 0.963 [0.937 to 0.991], respectively), while it increased the risk for GC (1.098 [1.059 to 1.139]).

**Table 5:** A multivariate analysis of the risk for CAG, PUD and GC based on age and the status of all five genes

Disease	Parameter	OR^d^	95 % CI^e^	*p* value
CAG^a^	*jhp0945*	0.214	0.099–0.466	0.0001
	*jhp0949*	0.373	0.172–0.807	0.0123
	age	0.964	0.937–0.992	0.0111
PUD^b^	*jhp0951*	2.821	1.224–6.499	0.0149
	age	0.963	0.937–0.991	0.0089
GC^c^	*jhp0945*	3.460	1.351–8.865	0.0097
	age	1.098	1.059–1.139	<0.0001

^a^*CAG* chronic active gastritis

^b^*PUD* peptic ulcer disease

^c^*GC* gastric carcinoma

^d^*OR* odds ratio

^e^*CI* confidence interval

## Discussion

The plasticity region is a recently identified locus found in the chromosome of *H. pylori* strains J99 and 26695 that displays similar characteristics to pathogenicity islands [7, 8]. The majority of the *H. pylori* strain-specific genes that are transferred from other species are located in the plasticity region [9, 12, 13]. The genes present in the plasticity region have been highlighted as potential pathogenic markers and may account for the differences in the *H. pylori* strain virulence, resulting in various clinical outcomes.

The *jhp0940*, *jhp0945*, *jhp0947*, *jhp0949* and *jhp0951* genes, which are specific for strain J99, have recently been reported to be associated with *H. pylori*-related diseases and are potential markers for the risk of gastrointestinal diseases [10]. Because of their geographic variation, the relationship between these genes and the severity of certain diseases has been discussed, but the results are often not in agreement. The prevalence of these genes and their relationship with certain diseases in China is currently unknown; therefore, we examined the distribution of these virulence markers and their relationship to the clinical outcomes of patients infected with *H. pylori.*

Previous study found that there were no significant associations between the gastroduodenal diseases and the status of *jhp0940*, *jhp0945* and *jhp0949* in East Asia strains [10]. Yakoob *et al.* demonstrated that *jhp0940* and *jhp0947* in Pakistan strains were associated with GC and PUD [14]. In our study, there was no association between *jhp0940* and diseases. The positive rates of *jhp0945*, *jhp0947, jhp0949* and *jhp0951* were much higher in PUD, and there was a significant association between the genes. However, the multivariate analysis showed that only *jhp0951* was independently associated with the development of PUD in our study. *Jhp0951*, which encodes an integrase from the XerCD family, is involved in the response to acidic environments [15]. In DUs, acid secretion increases, causing the mucosa to be continuously exposed to a low pH, which is consistent with our results [9]. As the virulence genes were associated with each other in our study, the strong linkage of *jhp0945*, *jhp0947* and *jhp0949* with PUD may be due to the significant association between *jhp0951* and PUD. We also speculate that all of these factors act synergistically in causing damage to the host.

It is well known that the development of GC is marked by a slow progression that begins with *H. pylori*-induced chronic superficial gastritis, which then progresses to atrophic gastritis, intestinal metaplasia, dysplasia and eventually GC [16, 17]. In our study, the multivariate analysis showed that the only independent virulence gene that increased the risk of GC was *jhp0945*, indicating a significant association between the two. However, the *jhp0945* status showed a negative association with CAG (Table 5), which conflicts with the process of GC development. In our study, we speculate that the majority of the individuals with CAG do not carry the *jhp0945* gene, while the few *jhp0945-*positive individuals potentially develop GC due to a combination of the bacteria, the host and other environmental factors, which is consistent with the fact that of the *H. pylori*-infected individuals, 80–90 % have clinically asymptomatic gastritis, while only 1–2 % develop GC [18]. *Jhp0945*-positive isolates may be more likely to develop severe diseases, and *jhp0945* status may be a risk indicator for GC development. Additional prospective studies are still necessary to further confirm our speculations.

## Conclusions

In conclusion, this was the first study in China to evaluate the relationship between plasticity region genes and clinical disease outcomes. We found that *jhp0951* status is an independent factor for discriminating PUD and may influence the association of other virulence factors with certain diseases. *Jhp0945* may predict the future development of GC in patients with CAG and is considered to be the best candidate disease marker for *H. pylori*-related diseases.

## Abbreviations

*H. pylori*: *Helicobacter pylori*
CAG: Chronic active gastritis
PUD: Peptic ulcer disease
GC: Gastric carcinoma
HLJ: Heilongjiang
JX: Jiangxi
OR: Odds ratio
CI: Confidence interval
